# Pharmaceutical industry, non-communicable diseases and partnerships: More questions than answers

**DOI:** 10.7189/jogh.07.020301

**Published:** 2017-12

**Authors:** David Beran, Margaret Ewen, François Chappuis, Tim Reed, Hans Hogerzeil

**Affiliations:** 1 Division of Tropical and Humanitarian Medicine, Geneva University Hospitals and University of Geneva, Geneva, Switzerland; 2 Health Action International, Amsterdam, the Netherlands; 3 Global Health Unit, Department of Health Sciences, University Medical Centre Groningen, Netherlands

When 21 biopharmaceutical companies recently launched the Access Accelerated Initiative (AAI) on prevention and care for non-communicable diseases (NCD) [1] in Davos, Switzerland, it was described as a ‘global, multi-stakeholder collaboration’. The need for partnership is included in the Sustainable Development Goals (SDG). SDG 17 aims to “Revitalize the global partnership for sustainable development” and requires inclusive partnerships between governments, civil society and the private sector [2].

The launch of the AAI leaves many questions unanswered regarding exactly how this partnership model is organized. From the little information publicly available, AAI presents a skewed view of what partnerships in global health should be. The International Federation of Pharmaceutical Manufacturers and Associations (IFPMA) acts as its Secretariat and no government or multi-lateral agency, besides the World Bank, is included. The Union for International Cancer Control (UICC) represents civil society. The AAI states that other NCD organizations will be involved, but these organizations are dwarfed by pharmaceutical company partners and most, including the UICC, are reliant on funding from these same companies [3].

Partnerships with pharmaceutical companies have shown some success, as seen with HIV/AIDS, vaccines and Neglected Tropical Diseases (NTD) [4-7]. In these partnerships each stakeholder had a specific role. Funding came from bi-lateral and multi-lateral donors, civil society played the role of advocate and implementer, and the pharmaceutical industry either developed new products or made existing medicines available for free or at differential prices with long-term pledges.

Responsibilities in addressing access to NCD medicines lie with the World Health Organization (WHO), governments, donors, and civil society. The role of the private sector is to complement these roles; not to replace them. Transparency in company initiatives is often lacking. It is therefore welcome news that AAI will be evaluated by the Boston University School of Public Health. However, this model of partnership raises the following questions:

1. What is the exact agenda in terms of diseases, approaches and countries, with stated objectives, baseline data and targets? How has this agenda been set and who has been involved?
2. Have the intended beneficiaries (patients, governments) been consulted and are locally available structures (such as national treatment guidelines) respected and supported? In general, are the WHO Guidelines for Drug Donations followed? Which partners are responsible for the various components of the program?
3. How will the AAI interact with a variety of other stakeholders, nationally and globally?
4. How will long-term sustainability be achieved?
5. How will accountability to the beneficiaries (patients, governments) be ensured?

NCDs are an unprecedented challenge globally. Universal access to NCD medicines requires long-term investment by all stakeholders, including companies. Long-term sustainability through locally available structures and resources should be a guiding principle in all phases of such initiatives. The SDGs and WHO’s Framework of Engagement with Non-State Actors, see an active role for private sector engagement. However, any partnership with the private sector needs to be framed within strict rules of engagement to avoid any perceived or real conflict of interest [8], and need to publicly address the fundamental questions we propose.

**Figure:**
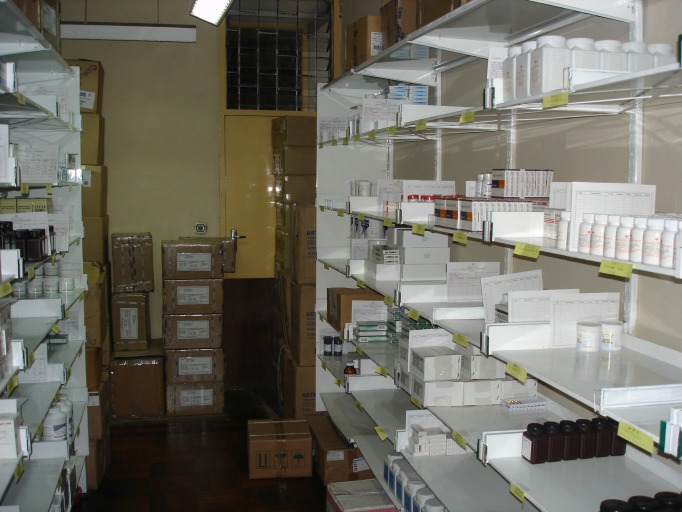
Photo: from David Beran’s own collection.
